# Parent-Implemented Interventions for Children with Special Needs in Türkiye: An Analysis of Single-Subject Research Studies

**DOI:** 10.3390/bs14121211

**Published:** 2024-12-17

**Authors:** Nesrin Sönmez, Serpil Alptekin

**Affiliations:** 1 Deparment of Special Education, Akdeniz University, Antalya 07058, Türkiye; nsonmez@akdeniz.edu.tr; 2 Deparment of Special Education, Ondokuz Mayıs University, Kurupelit Campus, Samsun 55200, Türkiye

**Keywords:** parent training, children with special needs, parent-implemented intervention, review

## Abstract

This article summarizes single-subject research studies that investigated the impact of interventions implemented by parents or family members of children with special needs living in Türkiye. In this study, 22 research studies conducted between 2013 and 2023 were analyzed in terms of their participants, methodological characteristics, characteristics of the training program implemented, and outcomes. Most of the child participants were boys, and most of the children were of school age. Mothers dominated parental involvement, but siblings also played an important role. The most frequently used design in the research methods was a multiple probe across participants design. While intervention fidelity data are reported in many research studies, implementation fidelity data are often omitted. The outcomes of the reviewed research show that parents successfully implemented the intervention and taught target skills to their children after the intervention they performed. However, generalization and maintenance findings were limited. The lack of clear reporting of parent training procedures makes it difficult to draw inferences about the effectiveness of the training. In general, although there is methodological diversity in the research reviewed, there is a need to be more rigorous about the clarity of the processes.

## 1. Introduction

Türkiye ranks 18th among 194 countries worldwide in terms of population size, with a total population of 85,279,553. It has a child population rate of 26.0%, which is higher than the child population rates of European Union (EU) member states [1]. Given the large population, challenges in regulating the education system are inevitable, and these challenges are even more pronounced for children with special needs (CSNs), who require tailored support and resources to access quality education.

Türkiye has made significant structural advancements in education, health, social life, and legal regulations within both international and national contexts. As a signatory to key international agreements, such as the Universal Declaration of Human Rights and the Declaration of the Rights of Persons with Disabilities, Türkiye guarantees the right to education under its constitution. With the enactment of Decree-law No. 573 in 1997 and subsequent regulations, significant reforms in the education of CSNs were introduced, including mandatory preschool education, individualized education programs, and the right for families to appeal diagnoses and placements [2]. However, while the regulation emphasizes family education and early education services, it lacks clarity on the planning, execution, and monitoring of these services. The absence of regulations for early intervention, team approaches, and family-centered practices remains a critical limitation.

According to OECD [3] data, teachers in many countries, including those in the developed world, require additional training to effectively teach CSNs. This highlights a critical issue: even when CSNs are included in educational settings, they may not fully benefit from quality educational opportunities. Consequently, family training practices emerge as a crucial strategy for enhancing these opportunities and supporting the education of CSNs.

The role of families in supporting the academic, social, and emotional development of individuals with special needs is well established. For families, particularly parents, to meet their responsibilities effectively, it is essential to provide them with the necessary services. These services may encompass a range of interventions, including training and support in various domains. They may also include psychosocial support and family-centered interventions, where parents learn to implement an intervention for their child [4]. To provide parents or family members with the knowledge and skills required to support their children’s development, family-centered interventions are referred to as ‘parent training’ [4], ‘parent coaching’ [5], ‘parent-mediated intervention’ [6], or ‘parent-implemented intervention’ [7].

In contrast to information-based parent training, family-centered interventions entail the training of parents or family members through one-to-one interactions to enable them to teach the child within the context of a program [4,8]. The defining feature of these interventions is that they entail interactions between the expert, the parent, and the child in a triadic configuration. In this study, the term ‘Parent-Implemented Intervention’ (PII) is employed because of a comprehensive review of all parent education programs, interventions, and coaching practices in which parents or family members assume the role of ‘interveners’.

PII involves teaching individuals in the family, commonly primary parents, to use a specific technique to help their CSNs acquire a specific target behavior through triadic (expert–parent–child) interactions [9,10]. Before the intervention, the presentation of some preliminary information within the framework of clinician–parent interaction takes place through training, including various methods and techniques. The necessary skills related to the intervention are provided to the parent practically through expert–parent–child interactions. In other words, in such interventions, while experts are the guides, parents are the implementers of the intervention. With PII, mothers [5,11,12], fathers [13], siblings [14,15], and other carers [16] can learn the techniques of the intervention and apply them reliably. A number of studies have demonstrated that parents can effectively teach their children a range of skills, including spontaneous imitation [17], social interaction [18], communication [19,20], and behavior modification [5], through the application of intervention strategies that they learned. These results indicate that parents and family members can serve as highly effective interventionists.

Several studies have been conducted to review the large body of literature on this topic. In many of the review studies on PII [6,8,21,22,23], subject research design (SSRD) was used in a significant portion of the studies included in the review. SSRD enables researchers to ascertain changes in individual performance through repeated measures [24]. In SSRD research, it becomes possible to establish a cause–effect relationship through repeated measurements before, during, and after the intervention [25]. These advantages frequently lead researchers to use SSRD in PII research as well as in clinician-implemented interventions. Despite this widespread use and well-documented research, there is a very limited review study focusing on SSRD investigating the effects of PII. Patterson et al. [26] conducted a review of SSRD studies that tested the impact of training programs for the parents of children with autism spectrum disorders (ASD). The study concentrated on a particular disability group (ASD) and a dependent variable (communication and social development). Additionally, Rakap and Rakap [27] investigated the efficacy of naturalistic language interventions conducted by parents of children with language impairments aged between 18 and 60 months.

The current review focused on studies conducted in Türkiye that investigated the effects of interventions implemented by parents or family members of CSNs using a SSRD, without being limited to any diagnostic group or intervention strategy. One reason for this was that more than 10 years had passed since the last review study [23] in Türkiye. Karasu and Tavil [23] conducted a review and meta-analysis that focused on providing parents with effective teaching methods. In this 2013 review, the researchers did not set a time limit. The review included nine studies conducted between 1999 and 2009, the majority of which used the SSRD methodology. In addition, the review included pre-test–post-test studies that used quasi-experimental control groups. The researchers examined and summarized the included studies in terms of independent and dependent variables, research designs, instruments, participants, and outcomes. They also calculated the effect sizes of the interventions used. It is evident that in the years since Karasu and Tavil’s [23] study, parent education studies in Türkiye have gained importance. It is noted that the significant increase in the number of studies on PII necessitated a new review. Furthermore, the inclusion of studies with group experimental designs in addition to single-subject studies in Karasu and Tavil’s [23] study was another reason for us to conduct this study. In another recent review, a descriptive review was conducted of studies that focused on parent education programs in Türkiye, including the gray literature, without any date limitation [28]. The researchers included only the research published in Turkish and excluded those published in English. This is considered an important limitation of this review.

The present study was designed to provide a comprehensive review of the current literature, considering the limitations of previous research. The objective was to provide transparency regarding the characteristics, variations in implementation procedures, and outcomes of the PIIs whose effects were examined using SSRD. CSN research in the Turkish context has gained significant international visibility and recognition for its quality. Türkiye is ranked among the top 10 countries with the highest number of SSCI-indexed publications in this field [29]. This highlights the country’s growing contribution to the global special education literature, making a systematic review of PIIs within this context particularly relevant for an international audience. It could be beneficial to examine SSRD on PIIs in Türkiye over the past decade (2013–2023) to gain insight into the current situation and to make recommendations for the future. Furthermore, we anticipated that the findings would assist in the identification of effective educational strategies for families of CSNs. The general aim of this study is to describe the characteristics of participants, the methodological features, and the basic outcomes of PIIs. In this study, we sought answers to the following questions regarding PII conducted with an SSRD in the last 10 years in Türkiye:

(a) What are the participant demographics of PII research?
(b) What are the methodological characteristics of PII research?
(c) What are the characteristics of the parent education procedures implemented in PII research?
(d) What are the main outcomes of PII research?

## 2. Materials and Methods

A literature review was conducted to outline the SSRD research on PIIs for CSNs in Türkiye. The review was conducted in accordance with the Preferred Reporting Items for Systematic Reviews and Meta-analyses (PRISMA) guidelines, as outlined in the PRISMA statement [30].

### 2.1. Search

In this study, we focused only on PII that took place in Türkiye. The main reason for this is to provide an original source for researchers in other countries by detailing the methods used and the results obtained in the context of Türkiye. We have designed a search strategy that we believe can provide guidance, particularly for countries with similar socio-cultural structures. We searched for articles in English and Turkish, published in Türkiye and abroad, that investigated the impact of PIIs in the education of SCNs between 2013 and 2023. In the search conducted in December 2023, the words “aile eğitimi” or “ebeveyn eğitimi” or “anne-baba eğitimi” or “anne”, “baba”, “kardeş” and “özel eğitim” or “özel gereksinim” or “engelli” were used for Turkish articles. To find English articles, the words “family training” or “parent training” or “parent”, “mother”, “father”, “sibling”, “family” and “special education” or “special needs” or “disabled” and “Türkiye” or “Turkey” were entered by trying different combinations. Articles were searched in different national and international electronic databases, such as APA, PsycINFO, TR Index, and Web of Science. Peer-reviewed academic journals were selected as the source type. Turkish word combinations were searched in the abstracts of the articles because it was aimed at collecting as many articles as possible.

English combinations of search words were searched only in “keywords”; however, it included the words “Türkiye” or “Turkey” in the combination each time so that it was searched “in all fields of the article”. The authors searched independently of each other in the libraries of their own universities. They then quickly skimmed the titles, and when that was not enough, the abstracts of all the papers were found, and they recorded the publication imprint information of the papers that could be reviewed in their library accounts. The authors then came together and merged the information on the articles found in this initial search into a single file. They conducted another search on the relevant search engines to find articles for which they did not have access to the full text. After this check, if there were still articles whose full text could not be accessed, they recorded them in a separate file. The authors also identified duplicate articles and deleted the latter from the list.

### 2.2. Screening and Selection of Eligible

When the list of articles for which full text was available was finalized in a single file, this file was saved as a Drive file, and the authors reviewed the titles of the articles in this list. At this stage, the authors carried out a review according to the following criteria to select appropriate studies: (a) they were related to the education of parents or family members with children with special needs, (b) they were conducted during 2013–2023, and (c) they included a PII. At this stage, the authors independently coded the studies that did not meet these three criteria by indicating which criteria they did not meet [(a) (b) (c)]. These codes were then transferred to an Excel file, and the inter-rater agreement was calculated by dividing the number of agreements by the sum of agreements by disagreements and multiplying this ratio by 100. At this stage, the inter-rater agreement was 100 percent.

### 2.3. Inclusion and Exclusion

In the third stage of the review, a more detailed review was performed according to the inclusion criteria. At this stage, the abstracts of the studies were analyzed, and for data that could not be accessed from the abstract, the whole article was read. These criteria were as follows:

(a) The participants were parents or family members of CSNs living in Türkiye;
(b) It was an empirical study published in peer-reviewed journals;
(c) The study was conducted with a SSRD.

Exclusion criteria include the following:

(a) Research involving interventions implemented by people other than parents or family members (such as teachers or clinicians);
(b) Parent training aimed at empowering individuals in various aspects (e.g., engagement, stress, anxiety, burnout);
(c) Research conducted with parents of typically developing children;
(d) Research involving families from different countries (including Türkiye);
(e) Review studies, surveys, or qualitative research;
(f) Intervention studies conducted with designs other than single-subject research designs (group experimental, action research, or mixed research designs);
(g) Research conducted with A–B design.

At this stage, the authors independently coded all studies to ensure inter-rater agreement. Inter-rater agreement was calculated using the same methods described in the second stage. Inter-rater agreement was 94% at this stage. The authors discussed and compromised for the studies that could not reach a consensus and finalized the list. The first author reviewed the references of the studies in this list to capture more at this stage. As a result of this review, it was determined that 5 more studies could be added to the existing list that met the inclusion criteria.

The descriptive characteristics of the studies that met the inclusion criteria were transferred to an Excel table. In addition to the titles of the relevant research (journal name, publication year, access address, DOI, publication title, author(s), etc.), the table included the following headings: (a) purpose; (b) research design; (c) dependent variable(s); (d) independent variable(s); (e) participants; (f) parent training procedure; (g) duration of the training; (h) format of the training; (i) setting of the training; and (j) results. The researchers entered the information independently of each other, and then the inter-rater agreement was calculated. The agreement between the first and second authors was 92% at this stage. The authors discussed points of disagreement until they reached 100% agreement.

## 3. Results

### 3.1. Study Selection

**Figure 1:**
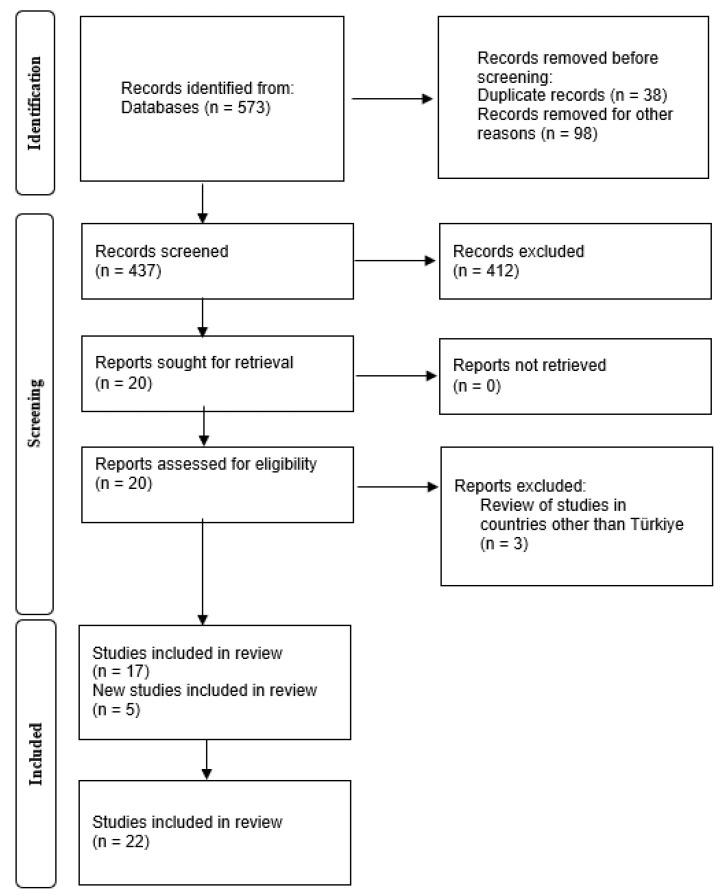
Prisma flow diagram showing search and screening for the review.

In the first searches made with combinations of Turkish and English search words in the databases, the years 2013–2023 were added to the filter, and articles published in peer-reviewed journals were also filtered so that research reports, book chapters, etc., were excluded. With the keywords entered and these filters applied, a total of 573 studies were collected in the first phase. The titles of the accessed studies were quickly scanned. Studies containing words such as stress, burnout, etc., in the title and conducted in fields other than education of CSNs (such as psychology, psychiatry, and sociology) were excluded from the list at this stage (n = 98). In addition, duplicate publications were identified and deleted (n = 38). After applying the criteria in Stage 2, 20 studies were obtained as potential candidates for inclusion. After detailed scrutiny according to the inclusion and exclusion criteria, three studies were excluded at this stage. There was a total of 17 research studies included, and, with the addition of 5 studies collected in the subsequent reference survey, a total of 22 studies were included in the review (as shown in Figure 1). The studies reviewed in the sample are included in the reference list.

### 3.2. Participants

Participants were analyzed separately for parents or family members and children. The characteristics of child participants in the studies were analyzed in terms of number, age, gender, and diagnostic characteristics. According to Table 1, the studies analyzed were conducted with a total of 57 children, including 48 boys and 9 girls. The ages of the children ranged from 3 years and 2 months to 31 years. Participant children are mostly between the ages of 3 and 12. In 4 studies, the age of the children was 15 years and above. The diagnoses of the participant children were autism spectrum disorder (n = 31), intellectual disability (n = 12), visual impairment (n = 6), writing disability (n = 3), autism spectrum disorder + intellectual disability (n = 3), developmental disability (n = 1), and intellectual disability + mild hearing loss (n = 1).

The characteristics of the parent participants were analyzed in the categories of number, age, role, and education level. As seen in Table 1, the research was conducted with a total of 57 family members, including mothers (n = 40), siblings (n = 10), fathers (n = 5), aunts, and grandmothers (n = 1). When the ages of the parents or family members were analyzed, it was seen that mothers were aged 24–53, fathers were aged 38–55, siblings were aged 9–20, the aunt was 52, and the grandmother was 60. In two studies, the ages of the participant parents were not specified. Of the parents or family members, 16 were high school graduates, 10 were primary school graduates, 7 were university graduates, 7 were secondary school graduates, 4 were college graduates, and 1 was a basic degree graduate. In addition, two parents (mothers) attend university and three family members (siblings) attend primary school.

### 3.3. Methodological Feature

The methodological characteristics of the studies were examined in the categories of research design, dependent variable, fidelity, and social validity and are given in Table 2. The studies were conducted using a multiple probe across participants (n = 9), a multiple probe across behaviors (n = 7), a multiple baseline across participants (n = 3), a multiple baseline across behaviors (n = 1), A-B-A-B (n = 1), and adapted alternating treatments (n = 1) designs. In 10 of these studies, the dependent variable for both children and parents was explained, while in 11 of the studies, only the dependent variables for children were explained. In one study, the dependent variable for neither children nor parents was specified at all. When analyzed in terms of parents, it was seen that teaching skills with simultaneous prompting (n = 4), toilet training (n = 1), teaching social interaction skills (n = 1), using the mand-model procedure, and teaching self-care skills (n = 3) were identified as dependent variables. The dependent variables for child participants were the levels of children in toilet control (n = 1), social interaction (n = 3), social skills (n = 2), playing games (n = 2), independent movement (n = 2), word production, self-care (n = 3), leisure time (n = 1), early math (n = 1), and daily life (n = 2) skills. In three studies, the term ‘target skills’ was used for the dependent variable.

In these studies, visual support, simultaneous prompting, the mand-model procedure, social story, video model, mobile teaching software, the responsive teaching early intervention program (ETEÇOM), and the Portage mother education program were specified as independent variables. In four of the studies, the independent variable was not specified at all. As seen in Table 2, implementation fidelity (IF) data were collected in only 2 research studies, while treatment fidelity (TF) data were collected in 19 research studies. Inter-observer reliability (n = 17) and social validity (n = 18) data were collected in the reviewed research. The social validity data were obtained via interviews with parents or family members, written opinions (n = 2), a questionnaire (n = 1), and social comparison with peers, in addition to interviews (n = 1).

### 3.4. Educational Procedures for Parents or Family Members

The procedures applied to teach the implementation of an intervention to parents or family members were analyzed in the categories of format and setting, the subject/skill taught, the strategies used, and the number and duration of sessions. As seen in Table 3, format and setting were specified in all studies except one. In 19 of the studies, family training was provided in a one-to-one format; in 1 of the studies, they were provided in a group format, and in 1 of the studies they were provided in both one-to-one and group formats. Parent training was organized as home-based (n = 6), home- and institution-based (n = 4), institution-based (n = 8) and online (n = 3). In terms of the skills taught to the parents in the program, the studies included simultaneous prompting (n = 5), constant time delay (using video model; n = 1), graduated guidance (with video model, n = 1), least-to-most prompting (n = 2), video model (n = 2), social story (n = 2), incidental teaching (n = 1), pivotal response training (n = 1), applying ETEÇOM (n = 1), using POW-C Space strategy (n = 1), using Independent Life Education (ILE) software (n = 1), and modeling (n = 1).

The training given to parents or family members included strategies such as visual supports (e.g., videos), informative lectures or verbal presentations, modeling, role-playing, practice, performance feedback, feedback and correction, homework, and showing positive and negative examples. Education programs were conducted using one or more of these techniques together. As seen in Table 3, the number and duration of parent education sessions were not specified in six of the studies. The number of sessions in the specified studies was at least 2 and at most 24. It was also seen that although the number of sessions was specified in six studies, the duration of the session was not specified. In the specified studies, it was determined that a session lasted at least 40 min and, at most, 6 h.

### 3.5. Outcomes

The outcomes of the reviewed research were handled under the three categories of effectiveness, generalization, and maintenance, as described below.

#### 3.5.1. Effectiveness

In SSRD studies, change in target behavior is determined directly and through repeated measurements before, during, and after the intervention [51]. The effectiveness outcomes for parents or family members were reported in 12 of the reviewed studies, as presented in Table 4. In all these research studies, parents or family members achieved the objectives of the program. Only in one study was one of the objectives of the program (social story writing) not fully achieved. In 16 of the studies, results were given for child participants, and all children acquired the target skills.

#### 3.5.2. Generalization

Generalization, which is assessed in SSRD research, is used in the absence of direct training to examine how well a learner performs target behavior in the context of a stimulus [51]. Despite this, generalization data were collected in 10 of the studies. In these studies, generalization data were collected for parents or family members (n = 6) and children (n = 10). An analysis of the data revealed that all participants successfully generalized the skills they learned across various contexts, including settings, people, materials, and target skills. Specifically, generalization data collected from parents or other family members indicated that participants generalized the target skills to different settings and materials in three studies (n = 3), to different target skills in two studies (n = 2), and to different settings in one study (n = 1). Similarly, data collected from child participants demonstrated that they generalized the target skills to different settings in four studies (n = 4), to different settings and materials in three studies (n = 3), to different settings and people in two studies (n = 2), and to different settings, materials, people, and target skills in one study (n = 1).

#### 3.5.3. Maintenance

Maintenance of the behavior means that the change/effect in the behavior is permanent [51]. When the maintenance data were analyzed, it was seen that maintenance data were collected in 19 studies. In these studies, maintenance data were collected for parents or other family members (n = 4) and children (n = 16). When these data were analyzed, it was seen that all participants maintained the skills they learned after a certain period. Maintenance measurements were carried out mostly with children, with the shortest 1 week and the longest 14 weeks after the training. In one study, maintenance data were collected and shown in a graph, but no clear information about the duration was shared.

## 4. Discussion

Parents and family members of CSNs play an important role in supporting their children’s learning and development. In this study of 22 studies that used SSRD in Türkiye between 2013 and 2023, PIIs were examined. The majority of child participants in PII studies conducted in Türkiye were male, of school age, and diagnosed with ASD. PII implementers were primarily mothers and siblings. The most commonly used research method was the SSRD, specifically the “multiple probe across participants” model. PII outcomes were reported to be positive for both parents and children. However, parent training procedures implemented for PII were not well documented.

The predominance of male children in the studies analyzed aligns with the findings of previous review studies [21,27,52], which have similarly reported a higher representation of boys in research on ASD [53]. This trend can be attributed to both the higher prevalence of ASD in males [54] and certain sociocultural and biological factors influencing child development. We believe that the source of this interest in ASD is the rapid increase in the prevalence of ASD (1 in 36; [54]) and the increasing need for early intervention services. Previous research has suggested that these gender disparities may also be shaped by diagnostic practices, which have historically been less likely to identify ASD in girls [21]. However, as highlighted in these studies, more research focusing on girls with ASD is essential to gain a clearer understanding of these gender differences and their implications for both diagnosis and intervention. Expanding research in this area could help address the current gap in the literature and provide a more balanced perspective on the needs of children with ASD. The reviewed studies revealed that specific learning disabilities and multiple disabilities were the least studied groups. This trend aligns with findings from a previous review of research methods in the education of CSNs, which indicated that SSRDs are rarely utilized with children who have learning disabilities [55]. This gap highlights a critical need for further research to explore the underlying reasons behind this limited focus and to address the challenges in designing and implementing studies for these groups.

The majority of participants were school-age children in the analyzed studies, which may reflect several intertwined factors. The later age at which ASD is commonly diagnosed, often after the age of 3, likely narrows the focus of research to this group. Additionally, school-age children may be more accessible for researchers, both in terms of availability and the readiness of families to participate in interventions. Families of children in this age group might have reached a stage of acceptance that facilitates active engagement in intervention processes, distinguishing them from families of younger children who are still navigating the initial stages of diagnosis and adjustment. This trend also underscores systemic challenges in Türkiye, where early intervention services remain underdeveloped. Conducting research on PIIs within family-centered early intervention frameworks may involve significant logistical and methodological complexities compared to expert-implemented approaches. These include the need for intensive behavioral or interaction-based methods, which demand substantial parental involvement and resources. Addressing these gaps through targeted studies could shed light on how to enhance the inclusion of younger children and expand the reach of early interventions in Türkiye.

The significant representation of mothers as primary implementers of interventions reflects broader global caregiving dynamics, where mothers often assume the role of primary carers, transcending cultural and geographical boundaries [21,52,56,57]. This trend highlights persistent gender norms and caregiving expectations within families, raising important questions about how these roles influence the success and sustainability of parent-mediated interventions. An equally noteworthy observation is the involvement of siblings as interventionists, even surpassing fathers. This finding suggests that siblings, often overlooked in intervention planning, can play a significant and effective role in supporting their family members [5,11,12]. Their active participation not only broadens the scope of who can implement interventions but also emphasizes the adaptability and inclusivity of these approaches within the family context. The inclusion of extended family members, such as aunts and grandmothers, further underscores the versatility of family-centered interventions. These results align with evidence suggesting that a diverse range of family members can act as effective interventionists, provided they are given the necessary training and support [5,11,12].

Moreover, the wide variation in the ages and educational levels of participants demonstrates the flexibility and accessibility of parent-mediated interventions. The ability of parents and family members, regardless of age or education level, to successfully implement these interventions speaks to the adaptability of the methods used [58]. However, questions remain regarding the extent to which factors such as age or education level might influence the outcomes of such interventions. Addressing these gaps through future research could help optimize training and support mechanisms for diverse family demographics.

The analyzed studies were mostly conducted with multiple probe designs between participants and the least amount were conducted with the adaptive alternating treatments design. Unlike this finding, in a review study examining PIIs conducted with a SSRD, it was reported that multiple baseline across-subjects design was used the most [27], and multiple baseline designs were used in the other study [26]. Although most SSRDs are frequently used in PIIs, review studies on this subject are limited, and the characteristics of the studies included in the review differ. It is natural that the designs used differ depending on many variables, such as the purpose of the research, dependent and independent variables, and participant characteristics. Each design has different advantages compared to each other, and this may have played a role in the decision of the researchers. Perhaps it would have been useful to discuss the least common research designs here. However, the fact that the inclusion criteria were different in this review study and the two previous studies makes it difficult to further the discussion.

The operational definition of dependent and independent variables for the measurement of outcomes in research is one of the SSRD standards [59,60,61]. Although it was specified in most of the analyzed studies, there were studies in which the dependent and independent variable(s) were not specified at all. A similar finding was reported by Patterson et al. [26] in a systematic review of training programs for parents of children with ASD. Although we did not conduct a methodological quality assessment in this study, we took the standards that SSRDs should carry as data points. This finding draws attention to the fact that researchers should be more careful about quality standards in SSRD research.

While treatment fidelity and implementation fidelity are often used interchangeably [21], the distinction between the two carries significant implications for understanding the effectiveness of PIIs. Implementation fidelity focuses on the accuracy of the expert’s behaviors—such as those responsible for training parents—while treatment fidelity pertains to how precisely parents, as interventionists, implement the intervention strategies [56,57]. The limited reporting of implementation fidelity in the analyzed studies raises concerns about the ability to evaluate the effectiveness of parent education strategies. Except for one study, most reviewed research reported data related to intervention fidelity rather than implementation fidelity, mirroring findings from similar reviews [8,21,26,27,62]. This gap underscores the need for studies to provide data on implementation fidelity, as it is critical for identifying ways to optimize parent education methods. Without such data, it becomes challenging to draw conclusions about the factors that enhance or hinder the training process for parents.

Nevertheless, the reported intervention fidelity data suggest that parents successfully implemented the interventions, which aligns with findings from prior PII reviews [6,8,21,22,23,26]. These results indicate that, despite the lack of implementation fidelity data, parents demonstrated the capability to apply intervention strategies effectively. Moving forward, greater emphasis on capturing implementation fidelity will not only enrich our understanding of parent education processes but also contribute to developing more robust and tailored support systems for families.

When procedural information of the parent training, such as setting, the method of delivery, and presentation strategies, is presented in detail and clearly, it may be possible to identify the different effects of these procedures on parent and child behaviors [63] and for service providers to plan future trainings accordingly [52,62]. The lack of detailed reporting on parent education procedures in many studies creates challenges in understanding the potential variability in their effects. Without clear descriptions of strategies, session structures, or implementation processes, it becomes difficult to assess how parent training methods can be refined or adapted to different family needs. Similar concerns have been raised in the literature, highlighting the importance of comprehensive reporting for advancing evidence-based practices and drawing reliable conclusions about the effectiveness of these interventions [27,52,62,64,65,66]. In addition to researchers providing detailed reports on training processes, studies should also include comparative analyses of training elements to identify the most effective and efficient procedural components. Despite this necessity, research on how procedural features change the effect of the intervention is limited.

Parents or family members were able to implement interventions effectively and teach target skills to their children. While most studies reported effectiveness outcomes for parents, children, or both, generalization findings were limited, with only a subset addressing generalization for parents. Similarly, maintenance outcomes were scarcely reported. The limited inclusion of parental behaviors as dependent variables in the studies likely contributed to the insufficient reporting of results from the parents’ perspective. Highlighting outcomes specific to parents is crucial for understanding the effectiveness of educational strategies and their broader impact. However, the lack of clarity in training procedures poses a challenge; even when parental outcomes are reported, the absence of detailed descriptions hinders the ability to draw meaningful connections between the training methods and their results. Addressing this gap through more systematic reporting can strengthen the evidence base and improve the applicability of PII in diverse contexts.

## 5. Conclusions

This review contributes to the existing literature by analyzing SSRD studies that investigate PIIs and their effects on both parent and child outcomes. While this review provides useful insights, it also identifies several gaps and limitations that warrant attention in future research.

One methodological limitation is the scope of this study, as it relied on pre-determined search terms and access to full-text articles available through university libraries. This may have restricted the comprehensiveness of the findings, potentially leading to an underrepresentation of certain populations or intervention types. Future reviews should consider expanding the range of search terms (e.g., including familial roles such as “grandmother” or specific disability groups) and using diverse search engines to capture a broader dataset.

This review did not assess the quality of the included studies, nor did it calculate effect sizes. While this aligns with the study’s focus on descriptive analysis rather than systematic review or meta-analysis, future studies should integrate these methodologies to provide more robust conclusions about the effectiveness of specific interventions. For instance, conducting meta-analyses to assess the effect sizes of PIIs on target behaviors can help identify patterns across different populations and intervention strategies.

Another key finding relates to the methodological clarity in SSRD studies. The reviewed studies often lacked a clear distinction between implementation fidelity (accuracy of the trainer’s behaviors) and intervention fidelity (accuracy of the parent’s behaviors). This gap limits the ability to evaluate the effectiveness of parent training strategies. Establishing a standardized framework for defining and measuring these variables in SSRD studies would enhance comparability and replicability. Researchers should prioritize reporting fidelity data for both trainers and parents to better understand the factors influencing intervention outcomes.

Although the findings confirm the effectiveness of parents as interventionists, there is a critical need to explore which elements of parent training procedures are most impactful. The reviewed studies did not consistently detail parent training strategies, such as the number of sessions or their duration, making it difficult to determine the relationship between training approaches and outcomes. Addressing this gap would provide actionable insights for practitioners designing family-centered interventions.

This review’s broad inclusion criteria—covering various target skills, disability groups, and methodological designs—provide a comprehensive overview but also suggest areas for more focused inquiry. Future reviews could investigate PIIs targeting specific behaviors or populations, such as early childhood interventions or strategies for adolescents. Additionally, exploring methodological variations, such as the use of different SSRD designs or measurement tools, could yield valuable insights into optimizing intervention outcomes.

From the perspective of Türkiye, the findings of this review are particularly significant. In the Turkish context, parent-implemented interventions have gained attention as part of efforts to address gaps in early intervention services and support for families of CSNs. However, challenges such as limited access to resources, variability in parent education practices, and the underdevelopment of family-centered early intervention programs persist. Highlighting these barriers in international platforms is crucial to improving service delivery and fostering collaborations that address these systemic issues. Moreover, documenting the effectiveness of PIIs in Türkiye can provide a model for other regions with similar challenges, emphasizing the universal applicability of such interventions.

Finally, this study underscores the scarcity of systematic reviews examining PIIs implemented through SSRD. Given the existing evidence supporting parents as effective interventionists, future systematic reviews and meta-analyses could significantly enhance the field by identifying best practices and refining intervention frameworks. This approach would not only address existing gaps but also provide practical guidelines for educators, clinicians, and policymakers aiming to maximize the impact of family-centered interventions.

## Figures and Tables

**Table 1:** Participant characteristics.

Study	Children				Parent/Family Member			
	n	Age	Gender	Diag. *	n	Age	Role	Level of Education
Batu et al. [31]	4	7	M	ASD	4	33 35–40	Mo	2 HS 2 U
Özkubat and Töret [32]	3	7	M	ID	3	28 35 24	Mo	2 PS 1 HS
Batu [33]	3	4.5 4.5 6	2 M 1 F	DD	3	28 27 42	Mo	HS
Özen [34]	3	5 5 6	2 M 1 F	ASD	3	9 9 11	S	2, PS 1, PL
Olçay-Gül and Tekin-İftar [35]	3	12 12 6	M	ASD	3	36 18 19	2 Mo 1 S	1 C 2 HS
Besler and Kurt [36]	3	6 6 5	M	ASD	3	30–40	Mo	PS C U
Karabulut and Tavil [37]	1	11	M	ID	1	13	S	PS
Çotuk and Altunay Arslantekin [38]	4	9 7 11 10	3 M 1 F	VI	4	17 11 15 20	S	Un
Acar et al. [39]	3	7 6 10	M	ASD	3	33–45	Mo	2 C 1 PS
Cankaya and Kuzu [40]	4	16 16 21 19	3 M 1 F	ID	4	42 45 60	3 Mo 1 GM	2 SS 1 HS 1 Bas
Aktaş and Çiftçi-Tekinarslan [41]	3	4–7	M	ASD	3	4–7	Mo	2 HS 1 PS
Gürel-Selimoğlu and Özdemir [42]	3	6 4 3 years + 2 months	2 M 1 F	ASD	3	32–35	Mo	2 U 1 HS
Ulugöl and Cavkaytar [16]	1	22	M	ID	1	52	A	PS
Aydın and Cavkaytar [43]	1	7	F	ASD	1	38	Fa	PS
Keser and Cavkaytar [44]	3	10 11 9	2 M 1 F	ASD + ID	3	38 41 26	Mo	U PS SS
Bayrak [45]	1	10	M	ID + MHL	1	Un	S	SS
Öğülmüş [46]	3	10 11 12	3 M	WR	3	Un	Mo	2 US 1 HS
Polat and Altunay [47]	2	6 5	1 F 1 M	VI	2	34 43	Mo	BD HS
Kurtoğlu and Cavkaytar [48]	1	18	F	ID	1	42	Mo	SS
Bozkus-Genc and Yucesoy-Ozkan [49]	4	58 mos 61 mos 51 mos 49 mos	M	ASD	4	32 32 37 47	3 Mo 1 Fa	PS SS HS D
Günlü and Çakmak [13]	3	15 16 20	M	ASD	3	52 43 55	Fa	BD SS AD
Kılıç and Yıldız [50]	1	4 years + 5 months	M	ASD	1	38	Mo	HS

M: Male; F: Female. * The diagnosis was noted as in the original research. ASD: Autism Spectrum Disorder; ID: Intellectual Disability; DD: Developmental Disability; VI: Vision Impairment; MHL: Mild Hearing Loss; WR: Writing Difficulties; Un: Undefined; Mo: Mother; S: Sibling, GM: Grandmother; A: Aunt; Fa: Father; HS: High School; BD: Bachelor’s Degree; SS: Secondary School; AD: Associate Degree; D: Doctorate; PS: Primary School; U: University; US: University Student; Bas: Basic Degree; C: College.

**Table 2:** Methodological characteristics.

Study	Study Design	Dependent Variable(s)	Independent Variable(s)	Fidelity	Social Validity
Batu et al. [31]	Multiple-probe design across participants	Mothers’ implementation of simultaneous prompting instruction Children learn target skills	Simultaneous prompting teaching method taught to mothers through visual support	IOA: − IF: − TF *: + (* treatment reliability)	+ Interview with mothers
Özkubat and Töret [32]	Multiple-baseline design across participants	Level of mothers’ acquisition of daytime toilet control skills for their children Children’s level of daytime toilet control	A family education program developed and implemented to provide mothers with the necessary skills to help their children gain toilet control	IOA: + IF *: + TF *: + (* TF is used for both IF and TF data)	+ Interview with mothers
Batu [33]	Multiple-probe design across participants (repeated for 3 participants)	Percentage of children’s accuracy in target skills	Simultaneous prompting instruction with visual support	IOA: − IF: − TF *: + (* treatment reliability)	+ Interview with mothers
Özen [34]	Multiple-probe design across participants	Typically developing siblings’ skill levels to perform social interaction behaviors Levels of the child with ASD in the target skill	Sibling training program with strategies to facilitate social interaction skills during iPad play activities with siblings with ASD	IOA: + IF: − TF: +	+ Interview with mothers
Olçay-Gül and Tekin-İftar [35]	Multiple-probe design across participant dyads	Children’s level of social skills	Un	IOA: + IF: − TF *: + (* treatment integrity)	+ Social comparison Interview with family members
Besler and Kurt [36]	Multiple-probe design across participants	Ratio of children’s correct answers regarding the ability to build a Lego train	Video model intervention provided by mothers	IOA: + IF: − TF *: + (* treatment integrity)	+ Questionnaire administered to family members
Karabulut and Tavil [37]	Multiple-probe design across behaviors	Performance of realization of the rules of the 3-rule children’s game by students	Sibling-mediated teaching offered	IOA: + IF: − TF: +	-
Çotuk and Altunay Arslantekin [38]	Multiple-probe design across participants	Simultaneous prompting practices of teaching siblings Children with visual impairment learn mobility skills	Simultaneous prompting teaching method taught by siblings with typical development	IOA: + IF: − TF: +	+ Interview with family members and instructor siblings
Acar et al. [39]	Adapted alternating treatment	Children’s levels of social skills	Un	IOA: + IF: − TF: + (* treatment integrity)	+ Interview with mothers
Cankaya and Kuzu [40]	Multiple-probe design across participants	Children’s level in target skills	Applications made with mobile skills teaching software	IOA: − IF: − TF: −	-
Aktaş and Çiftçi-Tekinarslan [41]	Multiple-probe design across participants	Frequency of mothers’ correct use of the mand-model procedure Percentage of children producing the target words	Parent education program	IOA: + IF: + TF: −	+ Interview with mothers
Gürel-Selimoğlu and Özdemir [42]	Multiple-baseline design across participants	Level of children’s social interaction skills	ETEÇOM program	IOA: + IF *: + TF *: + (* TF is used for both IF and TF data)	-
Ulugöl and Cavkaytar [16]	Multiple-baseline design across behaviors	Learning the skills of using a vacuum cleaner, cleaning the toilet, and mopping the floor with a mop	Instruction carried out by the aunt	IOA: + IF: − TF: +	+ Interview with aunt
Aydın and Cavkaytar [43]	Multiple-baseline design across behaviors	Level of acquisition of early math skills by the child Father’s level of learning to apply simultaneous prompting instruction	Simultaneous prompting instruction Education program for father	IOA: + IF: − TF: +	+ Interview with father
Keser and Cavkaytar [44]	Multiple-probe design across participants	Children’s level of learning the skill of cutting paper with scissors	Parent Education Program for Teaching Leisure Time Skills (SEZEP)	IOA: + IF: − TF: +	+ Interview with mothers
Bayrak [45]	A-B-A-B	Un	Un	IOA: − IF: − TF: −	-
Öğülmüş [46]	Multiple-probe design across participants	The story-writing skill levels of the children	The POW + C-SPACE strategy, applied by the parents	IOA: − IF: − TF *: + (* treatment integrity)	+ Printed opinion
Polat and Altunay [47]	Multiple-probe design across participants	Mothers’ implementations of teaching with simultaneous prompting procedure Acquisition of independent movement skills of children	Simultaneous prompting teaching method taught to mothers online	IOA + IF: − TF: +	+ Printed opinion from both parents
Kurtoğlu and Cavkaytar [48]	Multiple-probe design across participants	The mother’s level of acquiring the ability to teach personal care skills to her child The level of the child's personal care skills	Developed parent education program Teaching activities carried out by the mother with her child in line with the program	IOA + IF: − TF: +	+ Interview with mother
Bozkus-Genc and Yucesoy-Ozkan [49]	Multiple-baseline design across participants	The percentage of parents’ implementation fidelity Scores for parents’ interaction behaviors	Un	IOA + IF: − TF *: + (* treatment integrity)	+ Interview with parents
Günlü and Çakmak [13]	Multiple-probe design across participants Multiple-probe design across behaviors	The level of fathers’ implementation of teaching steps for self-care skills Children’s level of self-care skills	Father education program	IOA + IF: − TF: +	+ Interview with parents
Kılıç and Yıldız [50]	Multiple-baseline design across behaviors	Children’s level of self-care skills	Individualized ‘Portage Supported Mother Education Program’	IOA + IF: − TF: +	+ Interview with mother

IOA: Interobserver Agreement; IF: Intervention Fidelity; TF: Treatment Fidelity; Un: Undefined.

**Table 3:** Program characteristics.

Study	Format and Setting	Target	Delivery Method	Number and Duration of Sessions
Batu et al. [31]	1-1/H-B	Teaching self-care skills (brushing your teeth, wiping your mouth with a napkin, hand washing) with simultaneous prompting	Visual support (video recordings)	1 meeting after visual support
Özkubat and Töret [32]	1-1/I-B	Knowledge of toilet control skills Skills to keep a dryness record and to teach toilet control to their child	Information, modeling, rehearsing behavior, and independent practice	9–13 sessions Each session is 1–1.5 h
Batu [33]	1-1/H-B	Teaching self-care (hand washing, eating pudding) skills to the child via simultaneous prompting	Verbal description/role modeling, visual support (video recordings)	Un
Özen [34]	1-1/H-B	Behaviors of teaching social interaction skills to the sibling (inviting the sibling to play; giving instructions; taking turns; using clues; reinforcing appropriate play behaviors)	Watching sample video clips Explaining every social interaction with iPad Feedback and reinforcement during sibling play	6 sessions (40 min each)
Olçay-Gül and Tekin-İftar [35]	G/I-B	Writing and implementing social stories for social skills (greetings, asking for permission)	Handbook presentation Modeling	1 session 6 h
Besler and Kurt [36]	1-1/H-I-B	Teaching game-playing skills (building a Lego train) through video modeling	Information about video modeling. Practicing. Giving examples, question–answer. Support with verbal, visual, and written materials. Feedback at every step	2 sessions 3 h each
Karabulut and Tavil [37]	1-1/I-B	Helping siblings learn the rules of the game through modeling	A short description of how to become a model	Un
Çotuk and Altunay Arslantekin [38]	1-1/H-B	Teaching mobility skills via simultaneous prompting	Presenting the teaching method, modeling positive and negative examples, and practicing by changing roles	Un
Acar et al. [39]	1-1/I-B	Writing and implementing social stories to address skills	No details about the training method	Un
Cankaya and Kuzu [40]	Un	Teaching daily life skills (making cheese omelets) to the child using ILE software	Explaining the software on a home visit and practicing using the software on researchers	Un
Aktaş and Çiftçi-Tekinarslan [41]	1-1/I-B	Using the mand-model procedure to teach language skills to the child	Presentation, video demonstration, and role-playing; preparation of sample training sessions. Feedback on the play activities organized by mothers	4 sessions
Gürel-Selimoğlu and Özdemir [42]	1-1/I-B	Improving the interaction behaviors of the child by applying the ETEÇOM program	Explaining the daily plan; modeling; guiding; creating a homework plan	24 sessions for all mothers Each session is 1.5 h
Ulugöl and Cavkaytar [16]	1-1/H-B	Teaching the child daily living skills using the least-to-most procedure	Introduction to handbook Observation, feedback, and correction	4 sessions. Each session is 1–3 h
Aydın and Cavkaytar [43]	1-1/H-I-B	Teaching math skills with simultaneous prompting to the child	Information, role-playing, modeling, performance feedback	1 session of 60 min (for 2nd stage)
Keser and Cavkaytar [44]	G+1-1/ H-I-B	To teach the child the skill of cutting paper with scissors by least-to-most procedure	Handbook; telephone communication; keeping a communication book	Information: 2 sessions, 1 h each Implication: 1 session, 2 h
Bayrak [45]	1-1/O	Teaching how to make a request with the incidental teaching method	Information Guiding the mother from a distance during the intervention	Un
Öğülmüş [46]	1-1/O	Using POW + C-SPACE strategy in story writing instruction	Videos of training sessions, sample stories, story rockets, graphic organizers, and teaching notes	3 sessions of 45 min each
Polat and Altunay [47]	1-1/O	To teach independent movement skills to the child by using the simultaneous prompting procedure	Presentation Showing positive and negative models with videos Sessions where mothers take on the role of teacher	2 sessions (presentation)
Kurtoğlu and Cavkaytar [48]	1-1/H-I-B	To teach self-care skills to the child by using the personal viewpoint technique	Presentation and modeling; handbook; home visits and telephone interviews	4 meetings at the institution 1 home visit (1.5 h)
Bozkus-Genc and Yucesoy-Ozkan [49]	1-1/H-I-B	Implementation of pivotal response training	Pivotal response training handbooks (6 units) Presentation and video; modeling; corrective feedback Homework; feedback to videos; observation and immediate feedback during home visits	A total of 12 sessions, each lasting 15 min–6 h
Günlü and Çakmak [13]	1-1/H-I-B	Teaching skills to children using constant time delay and video modeling	Presentation, practice, feedback, homework	5 sessions
Kılıç and Yıldız [50]	1-1/H-B	Teaching the child using graduated guidance with video model	Presentation, modeling, feedback on recorded videos; homework	10 weeks: 2 h home visits 2 days a week

Un: undefined; 1-1: one to one; G: group; H-B: home based; I-B: institution based; H-I-B: home and institution based; O: online.

**Table 4:** Outcomes.

Study	Effectiveness	Generalization	Maintenance
Batu et al. [31]	The simultaneous prompting procedure taught through the training CD prepared for mothers was found to be effective in teaching target skills to their children.	Generalization data could not be collected for one child. Other children and mothers generalized the target skills to different settings and materials.	+ Except for one participant, the children maintained the skills after 2 weeks.
Özkubat and Töret [32]	The implemented parent education program is effective in helping mothers gain the necessary processes related to the skills of keeping dryness records and gaining toilet control. Mother-implemented toilet training interventions resulted in the children acquiring toilet control skills.	-	+ All children maintained their toilet control skills after 2, 4, and 6 weeks.
Batu [33]	Mothers learned to use simultaneous prompting to teach chaining skills to their children. Children acquired the skills taught by their mothers at home.	All children and mothers generalized the target skills to different settings and materials.	+ The children maintained the target skills they acquired for 2 weeks.
Özen [34]	The sibling training program was found to be effective in teaching social interaction skills necessary for iPad play activities.	All the learner children generalized the social interaction skills they learned to different settings.	+ All children maintained the social interaction skills they learned after 2 weeks.
Olçay-Gül and Tekin-İftar [35]	After the training, the family members did not learn how to write a SS 100% accurately, but they learned how to implement the SS intervention reliability. Social stories provided by family members were found to be effective in children’s acquisition of target social skills.	Children who gained social skills through social stories presented by family members generalized the target skills they acquired to different environments and people.	+ Children maintained the target skills after 5 weeks.
Besler and Kurt [36]	Mothers achieved 100% success in preparing and implementing videos for skill teaching. Teaching with the video modeling made by the mothers was found to be effective in teaching children the skill of making a Lego train.	The mother generalized the video preparation skill to different skills with 100% success (data could not be collected because 1 mother left the study). Children generalized the skills they learned to different settings and people.	+ One of the mothers maintained her video preparation and implementation skills after 5 weeks (1 mother dropped out of the study and data could not be collected). Children (2 of them) maintained target skills after 28 days.
Karabulut and Tavil [37]	The sibling-mediated teaching was found to be effective in teaching game rules to the learner siblings.	-	+ The learner sibling acquired the rules of the game after 1, 3, and 4 weeks.
Çotuk and Altunay Arslantekin [38]	The instructor siblings implemented the practice sessions in a highly effective and reliable manner. There were significant differences in terms of the correct responses of the learner siblings in exhibiting independent mobility skills before and after the intervention.	All participants were able to generalize the skills they learned in a different setting.	+ Children maintained the skill after 35 days.
Acar et al. [39]	All mothers learned how to develop SS/VM and implemented the intervention with their children with 100% accuracy. Mother-administered SS and VM were found to be almost equally effective in teaching social skills to three children with ASD.	Mothers generalized the acquired skills to teach different behaviors. All children generalized the acquired target skills in different settings with 100% accuracy.	+ Children maintained the social skills they acquired 14 weeks later.
Cankaya and Kuzu [40]	Mobile skill-teaching software was found to be effective for individuals with intellectual disabilities after the skill-teaching activities were carried out.	-	+ Follow-up data were collected from the children and presented in the graph, but the duration was not reported.
Aktaş and Çiftçi-Tekinarslan [41]	The mand-model procedure used by mothers was found to be effective in teaching new words to children with ASD.	-	+ A follow-up was performed 2 weeks later to assess the children’s skills.
Gürel-Selimoğlu and Özdemir [42]	Children showed improvements in social interaction skills.	-	+ Children maintained their social interaction skills after two months by increasing them.
Ulugöl and Cavkaytar [16]	Auntie’s level of knowledge about the program increased. The child gained target skills.	-	-
Aydın and Cavkaytar [43]	The father’s simultaneous prompting instruction was found to be effective in the child’s acquisition of early math skills.	-	+ A follow-up was performed 3 weeks later to assess the child’s skills.
Keser and Cavkaytar [44]	Parents achieved the goals of the parent education program. Instruction with graduated guidance implemented by the parents was found to be effective in children’s acquisition of the skill of cutting paper with scissors.	-	-
Bayrak [45]	The incidental teaching method applied with distance parent education was found to be effective in gaining the ability to express requests.	-	-
Öğülmüş [46]	Mothers were able to implement the strategy reliably, and children acquired story-writing skills at the criterion level.	-	+ A follow-up was performed 5 weeks later to assess the children’s skills.
Polat and Altunay [47]	Teaching with simultaneous prompting through online mother teaching is effective in children’s acquisition of independent movement skills.	+ Children were able to generalize the independent movement skills they learned to different environments.	+ A follow-up was performed 1, 2, and 3 weeks later to assess the children’s skills.
Kurtoğlu and Cavkaytar [48]	The mother carried out the intervention process reliably; the intervention by the mother was effective in the child’s acquisition of self-care skills.	The child generalized different target skills to different environments, different people, and different target skills to tools and materials.	+ A follow-up was performed 1, 2, and 3 weeks later to assess the child’s skills.
Bozkus-Genc and Yucesoy-Ozkan [49]	All parents were able to perform the PRT with a high level of reliability.	-	+ A follow-up was performed 2, 4, and 6 weeks later to assess the parents' skills.
Günlü and Çakmak [13]	The father training program was found to be effective in teaching self-care skills to the children of the participant fathers.	-	+ A follow-up was performed 1, 3, and 5 weeks later to assess the children’s skills.
Kılıç and Yıldız [50]	The program had a positive effect on the child’s development of self-care skills, and skills were acquired.	Skills are generalized to different settings or materials.	+ A follow-up was performed 1, 3, and 5 weeks later to assess the child’s skills.

## Data Availability

The original contributions presented in this study are included in the article. Further inquiries can be directed to the corresponding author.
